# Mitochondrial ribosomal protein S18-2 evokes chromosomal instability and transforms primary rat skin fibroblasts

**DOI:** 10.18632/oncotarget.4123

**Published:** 2015-05-12

**Authors:** Suhas D. Darekar, Muhammad Mushtaq, Sreeharsha Gurrapu, Larysa Kovalevska, Catherine Drummond, Maria Petruchek, Luca Tirinato, Enzo Di Fabrizio, Ennio Carbone, Elena Kashuba

**Affiliations:** ^1^ Department of Microbiology, Tumor and Cell Biology (MTC), Karolinska Institutet, Stockholm, Sweden; ^2^ R.E. Kavetsky Institute of Experimental Pathology, Oncology and Radiobiology, NASU, Kiev, Ukraine; ^3^ King Abdullah University of Science and Technology, PSE and BESE Divisions, Thuwal, Kingdom of Saudi Arabia; ^4^ University “Magna Græcia” of Catanzaro, Viale Europa, Località Germaneto, Catanzaro, Italy

**Keywords:** MRPS18-2, cell transformation, mitochondrial ribosomal protein, rat skin fibroblasts, chromosomal instability

## Abstract

We have shown earlier that overexpression of the human mitochondrial ribosomal protein MRPS18-2 (S18-2) led to immortalization of primary rat embryonic fibroblasts. The derived cells expressed the embryonic stem cell markers, and cellular pathways that control cell proliferation, oxidative phosphorylation, cellular respiration, and other redox reactions were activated in the immortalized cells.

Here we report that, upon overexpression of S18-2 protein, primary rat skin fibroblasts underwent cell transformation. Cells passed more than 300 population doublings, and two out of three tested clones gave rise to tumors in experimental animals. Transformed cells showed anchorage-independent growth and loss of contact inhibition; they expressed epithelial markers, such as E-cadherin and β-catenin. Transformed cells showed increased telomerase activity, disturbance of the cell cycle, and chromosomal instability. Taken together, our data suggest that S18-2 is a newly identified oncoprotein that may be involved in cancerogenesis.

## INTRODUCTION

Understanding the molecular mechanisms underlying cell transformation is one of the most important and intricate challenges of modern cancer biology. *In vitro* and *in vivo* models have been developed to identify the molecular details of cell transformation. The first model created was the transformation of hamster cells upon simian virus 40 (simian vacuolating polyomavirus, SV40) infection [1]. In rare cases, the viral genome was integrated into the host genome of “nonpermissive” hamster cells and was transcribed along with normal cellular genes. The infected cells could not produce new viral particles but became transformed and lost normal control of cell growth (discussed in [2]). In this model, a few characteristics distinguished transformed cells from primary adherent cells: the cells changed morphologically, became immortal, lost contact inhibition, and acquired the ability for anchorage-independent growth. All of these features were easy to monitor because the cells overcame the Hayflick limit of division (see [3]), formed foci in the culture and in soft agar, and gave rise to tumors in animals.

Subsequently, primary cells of different origins (human, monkey, hamster, mouse, rat, etc.) have been induced to transform *in vitro*. The molecular mechanisms responsible for cell transformation by DNA tumor viruses include general inactivation of the p53 (TP53, NP_001119584) and retinoblastoma protein (RB, NP_000312) pathways (reviewed in [2]). For example, upon SV40-induced transformation, viral large T protein binds to the DNA-binding domain of p53, inactivating the transcriptional activity of the latter [4], and to RB [5], disrupting the repressive RB–E2F1 complex, that liberates E2F1 (NP_005216) and promotes S phase entry.

We have reported earlier that the mitochondrial ribosomal protein MRPS18-2 (S18-2, NP_054765) binds to RB [6] and prevents the formation of the E2F1–RB complex that leads to elevated levels of free E2F1 protein in the nucleus and the subsequent promotion of S phase entry [7]. Moreover, overexpression of S18-2 in primary rat embryonic fibroblasts (REFs) resulted in their immortalization with a stem cell phenotype [8]. We have found that 4209 genes and at least 19 cellular pathways were altered in the S18-2-immortalized cells [9]. Genes involved in regulation of proliferation, oxidative phosphorylation, cellular respiration, and other redox reactions were upregulated in immortalized 18IM cells, which became more active metabolically. Despite these changes in the gene-expression profile and the increased metabolic activity, 18IM cells were unable to produce tumors in SCID mice, and they transdifferentiated [8].

Here, we report that primary rat skin fibroblasts (RSFs) undergo malignant transformation upon overexpression of S18-2 protein. Cells passed more than 300 population doublings, lost contact inhibition, and could give rise to tumors in experimental animals.

## RESULTS

### Immortalization of primary RSFs upon overexpression of S18-2

**Figure 1:**
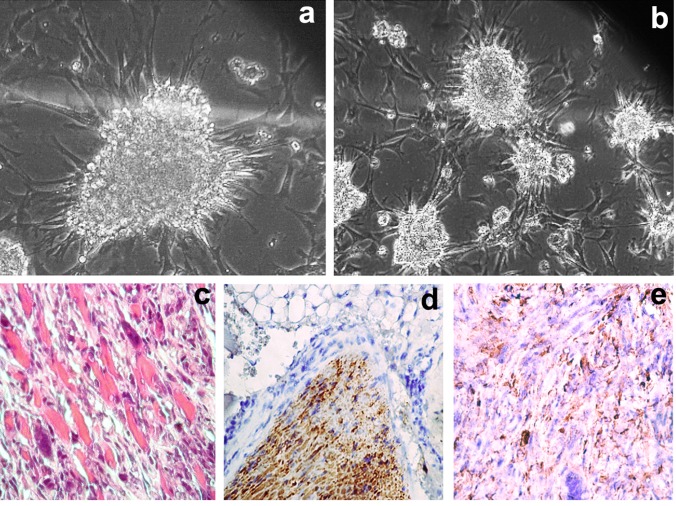
Growth pattern of clone 6, produced from RSFs upon GFP-S18-2 overexpression in bacterial petri dishes and in SCID mice Note that cell aggregates were attached to the surface of the bacterial petri dish **a.** and **b.** Tumors were classified as invasive fibrosarcomas after staining with hematoxylin and eosin **c.** note the mouse muscles invaded by the tumor. All tumor cells expressed S18-2, as shown by rabbit anti-S18-2 antibody **d.** Tumor cells retained vimentin expression **e.**, a characteristic of mesodermal cells.

As mentioned earlier, primary REFs turned into stem-like cells upon S18-2 overexpression. To determine whether this effect is common in other cells, 2–5×10^5^ primary RSFs (derived from a tail, passages 3–5) were grown in a 7.5-cm-diameter petri dish and transfected with a plasmid encoding the GFP-fused S18-2. Transfected cells were selected with 0.5 mg/ml G418. After 3 weeks, 18 of about 50 colonies obtained from four petri dishes were seeded into six-well plates. The fastest growing clones (numbers 3, 6, 13, and 17) were expanded in 50-ml culture flasks. The transfected cells lost contact inhibition and showed anchorage-independent growth, and these cells were cultured *in vitro* for more than 30 months and were passaged for more than 400 population doublings. All control RSFs became senescent and died after 4–5 weeks. Transformed cells could grow in a bacterial petri dish and formed foci on the attached cells (see growth of clone 6, Figure 1a and 1b).

To characterize the obtained immortal cells, their tumorigenicity was tested in experimental animals (SCID mice). RSFs immortalized by GFP–S18-2 (clones 6, 13, and 17) along with REFs immortalized by pBabe–S18-2 (clones 2, 4, and 6, reported in [9]) and by GFP–S18-2 (18IM and clones 12, 10 described in [8] and [9], respectively) were injected subcutaneously into mice (0.5–2×10^6^ cells per mouse, see Table 1).

**Table 1:** Immortalized cells gave rise of tumors in experimental animals

Cell lines		Number of mice	Tumor rate
Rat embryonic fibroblasts	18IM	4	0
	Clone 2, pBabe-S18-2	2	0
	Clone 4, pBabe-S18-2	2	0
	Clone 6, pBabe-S81-2	2	0
	Clone 10, GFP-S18-2	4	4 (100%)
	Clone 12, GFP-S18-2	4	0
Rat skin fibroblasts	Clone 6, GFP-S18-2	3	2 (67%)
	Clone 13, GFP-S18-2	3	0
	Clone 17, GFP-S18-2	3	0

18IM cells and immortalized REFs (clones 10 and 12) were inoculated into four animals for each cell line, and other clones were inoculated into two mice each. Each clone of the immortalized RSFs was introduced into three mice. Tumors were found in 100% (4/4) of the experimental animals after inoculation of clone 10 from REFs and in 67% (2/3) of mice after introducing clone 6 derived from RSFs. Tumors were detected 2 months after inoculation of immortalized REFs (clone 10) and 3 months after inoculation of immortalized RSFs (clone 6). Five months after inoculation, the mice that bore tumors developed cachexia, and the experiments were terminated. The tumors were characterized as aggressive invasive fibrosarcomas (Figure 1c). All tumor cells showed S18-2 staining (Figure 1d). The tumor cells were positive for both mesenchymal (smooth muscle actin (SMA), partially positive for vimentin; see Figure 1e) and epithelial (E-cadherin) cell markers, a feature of mesothelial tumors. Notably, the tumors formed by clone 6 of immortalized (or rather transformed) RSFs contained aneuploid cells.

### Comparative study of the expression patterns in primary, immortalized cells and in tumors at the mRNA and protein levels

**Figure 2:**
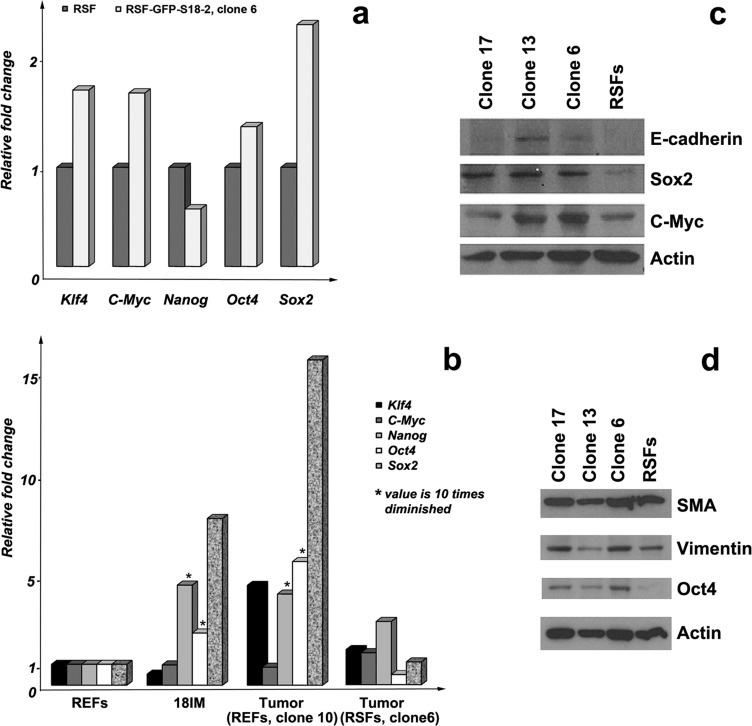
Gene-expression patterns in primary RSFs and immortalized cells mRNA expression was assessed by Q-PCR. Three or four reactions (each in triplicate) were run for each gene, and the standard deviation was calculated. Gene-expression pattern **a.** in primary RSF and immortalized cells (RSF–GFP–S18-2, clone 6) and in primary REFs, 18IM cells, and two of the tumors grown in experimental animals from REF–GFP–S18-2 clone 10 and RSF–GFP–s18-2 clone 6 **b.** * - the value is decreased by 10-fold in the figure. Protein expression levels were compared between parental RSFs and immortalized cells using Western blotting **c.**, **d.** Note that Sox2, Oct4, c-Myc, and E-cadherin were overexpressed in the immortal clones. SMA and vimentin were expressed in parental RSFs and in transformed clones.

To characterize the new cell lines, the expression patterns of several genes were studied by comparing the primary, immortalized cells and tumors. As mentioned above, expression of *Oct4*, *Sox2*, and *Nanog* genes, which contribute to the induction of pluripotency, was elevated in immortalized 18IM cells. This contrasted with *Klf4* and *c-Myc* expression, which was downregulated at the mRNA level (see [9]). Q-PCR was performed to investigate the expression of these genes in S18-2-immortalized adult RSFs and in two of the tumors obtained after inoculation with RSF–GFP–S18-2 (clone 6) and REF–GFP–S18-2 (clone 10). The expression of *Klf4, c-Myc, Oct4*, and *Sox2* was upregulated in immortalized cells compared with the parental RSFs (Figure 2a). Notably, a similar expression pattern was observed in the tumors produced from RSF–GFP–S18-2 clone 6 and REF–GFP–S18-2 clone 10 cells (Figure 2b). Importantly, *Klf4, Nanog, Oct4*, and *Sox2* genes were markedly upregulated in tumors produced from REF–GFP–S18-2 clone 10 cells, in contrast to 18IM, in which *Klf4* expression was downregulated compared with primary cells.

**Figure 3:**
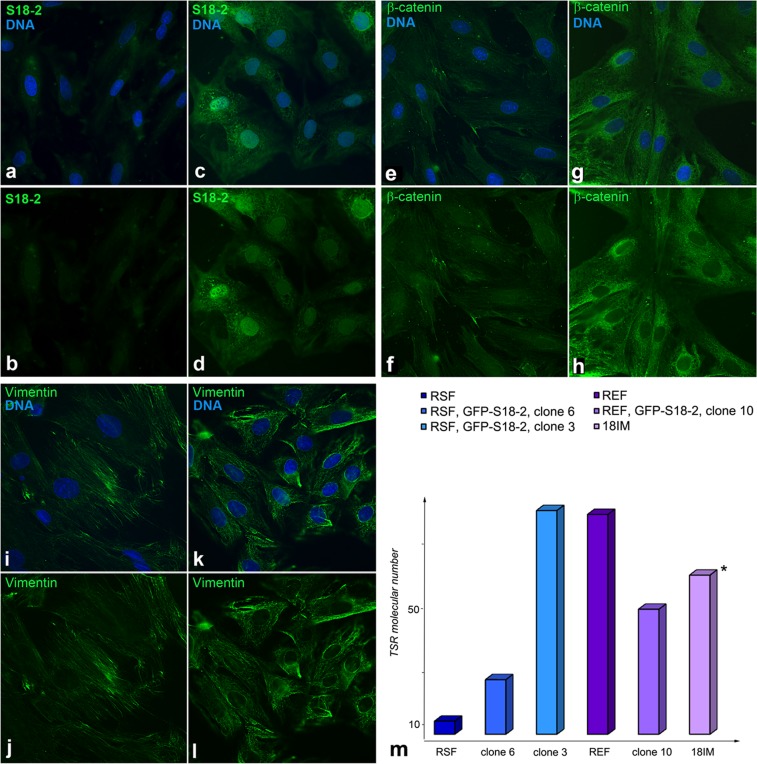
Comparison of primary and transformed cells Immunostaining produced a weak S18-2 signal (green) in the cytoplasm of RSFs (panels **a** and **b**) and a strong signal in the cytoplasm and the nucleus in transformed cells (clone 6) (panels **c** and **d**). DNA is shown in blue. The β-catenin signal (green) was observed in clone 6 (panels g and h) but not in the primary RSFs (panels **e** and **f**). Vimentin filaments (green) were evident in control RSFs (panels **i** and **j**) but were disturbed in cells of clone 6 (panels **k** and **l**). Telomerase activity was compared between the immortalized cells and the control primary REFs and RSFs (panel **m**). Greater activity of telomerase was detected in immortalized clones compared with parental cells. * - value is decreased by 10-fold in the figure.

The protein expression pattern was investigated by Western blotting and immunostaining in parallel with the study of mRNA expression. Importantly, Oct4 and Sox2 were induced in all clones of immortalized RSFs compared with the parental cells (Figure 2c and 2d). Vimentin and SMA were detected in both the clones and parental cells. c-Myc was highly expressed in clones 6 and 13. E-cadherin, which is usually expressed in epithelial cells, showed a signal in immortalized cells but not in primary RSFs (Figure 2c). The lack of E-cadherin expression in fibroblasts may suggest the presence of the mesenchymal–epithelial cell transition (MET). Immortalized cells, but not primary cells (Figure 3e and 3f), also showed a β-catenin signal (Figure 3g and 3h). This observation is also consistent with the presence of the MET.

As noted above, Western blot analysis showed that vimentin was expressed uniformly by both the immortalized cells and control primary cells (see Figure 2d). However, the vimentin fibers were unstructured in the immortalized clones (Figure 3k and 3l); this lack of structure is a sign of cell transformation. Notably, S18-2 was detected in both the cytoplasm and the nucleus of the transformed cells (Figure 3c and 3d), whereas primary RSFs showed only weak cytoplasmic S18-2 staining (Figure 3a and 3b).

Hence, the immortalized RSFs showed a lack of contact inhibition, elevated expression of *Klf4*, c-Myc, Oct4, and Sox-2, and the presence of epithelial cell markers, such as E-cadherin and β-catenin, and could form tumors in experimental animals. All of these changes indicated a transformation. We speculate that the elevated expression of *c-Myc* and *Klf4* was one of the causes of tumor formation.

### Telomerase activity in primary cells and transformed clones

To characterize the immortalized and transformed cells further, the telomerase enzymatic activity of the primary REFs and RSFs was compared with that of the 18IM cells, clone 10 from REFs, and clones 3 and 6 from RSFs. The number of telomere repeats added by telomerase was larger in immortalized cells (Figure 3m), especially in 18IM cells, compared with the terminally differentiated primary RSFs, which produced a minimal number of telomere repeats. Elevated telomerase activity is a prerequisite for transformation and is often associated with cell cycle distortion [10].

### Cell cycle analysis of primary and immortalized cells

The cell cycle of immortalized and primary cells was analyzed to determine whether S18-2 overexpression can influence the cell cycle. The cell cycle distribution of primary REFs and RSFs was determined and compared with that of cells expressing GFP–S18-2 constitutively (18IM cells, REF–GFP–S18-2 clone 10, and RSF–GFP–S18-2 clone 6).

**Figure 4:**
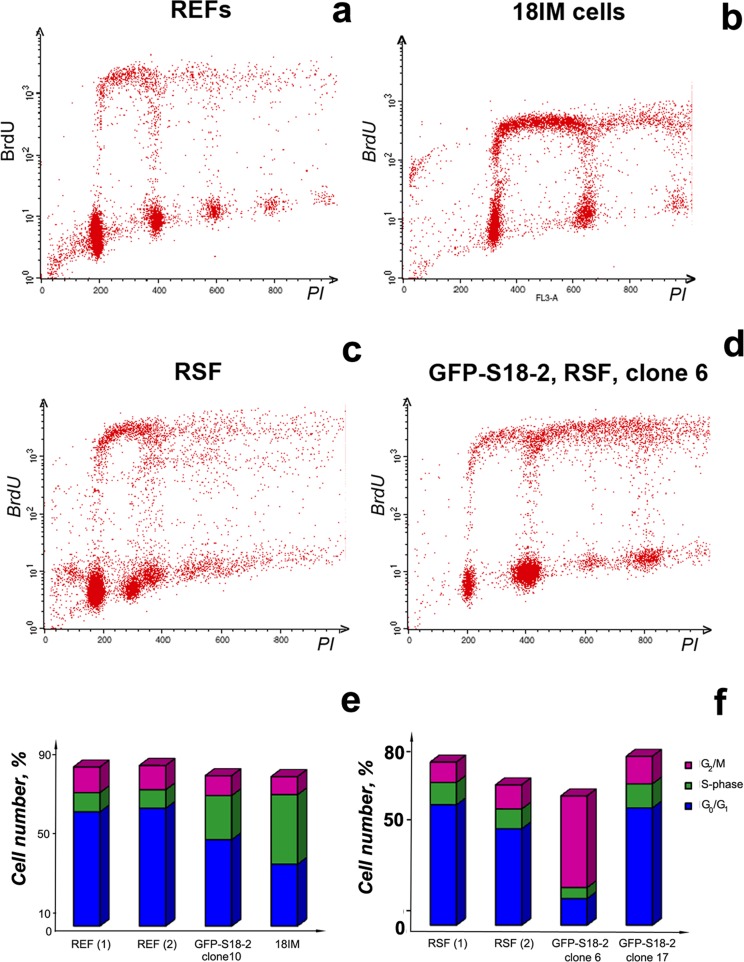
Cell cycle distribution of the immortalized 18IM cells, clone 6 (RSF–GFP–S18-2), and control primary REFs and RSFs DNA synthesis and DNA content were assessed by measuring bromodeoxyuridine (BrdU) incorporation and propidium iodide (PI) staining by flow cytometry. Populations were gated, and the relative proportions of G_1_, S, and G_2_/M phase cells were determined. Primary cells are presented in panels **a** and **c**, immortalized cells in panels **b** and **d**. Cell cycle distribution (as a percent of the total number of cells) are shown in panels **e** for REFs and **f** for RSFs.

Cells were grown to equal confluence (70–80%), stained with primary mouse monoclonal anti-BrdU- and secondary sheep anti-mouse FITC-conjugated antibodies, and then with 25 μg/ml propidium iodide. The stained cells were analyzed by flow cytometry (Figure 4). The RSF control cells appeared as a mixed population (Figure 4c), which was consistent with the origin of these cells. The S phase content differed significantly between immortalized cells and primary REFs: a higher proportion of 18IM and REF-S18-2 clone 10 cells were found in S phase compared with primary REFs (Figure 4a, 4b, and 4e). A corresponding reduction in the proportion of cells in G_1_ phase was also observed. By contrast, the proportions of immortalized and primary RSFs in S phase did not differ between cells (Figure 4c, 4d, and 4f). However, clone 6, which was tumorigenic in SCID mice, showed evidence of aneuploidy (Figure 4d), and a proportion of these cells had a DNA content of “8N”. The number of cells in G_2_/M was also significantly greater in clone 6 cells than in control cells; this difference might reflect a tetraploid G1 population (Figure 4f).

### Study of chromosomal instability (karyotyping)

Considering that aneuploidy was detected in both RSF clone 6 cells and in tumors that were obtained from this cell line, we performed karyotyping of clone 6 cells. For comparison, REFs transformed by overexpression of *C-MYC* and mutated *RAS* (*Ha-RAS*) genes and the control RSFs were also studied.

**Figure 5:**
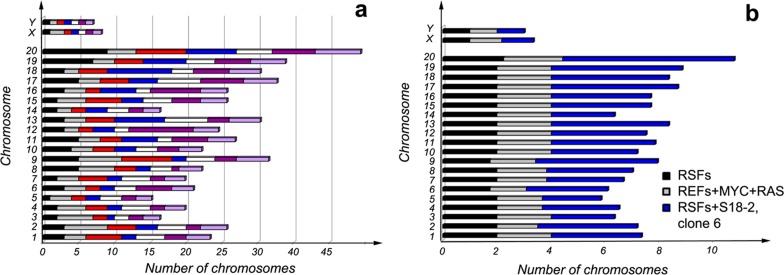
Karyotypes of primary RSFs, REFs transformed upon overexpression of C-MYC and Ha-RAS, and RSFs immortalized by S18-2 clone 6 Note that S18-2 induced chromosomal instability **a.** when each of the metaphases analyzed differed from the normal diploid number of chromosomes. Each metaphase is shown in a different color. The average number of each chromosome in clone 6 cells is presented in panel **b.** in comparison with *C-MYC* and *Ha-RAS* in transformed REFs and the control primary RSFs. At least five metaphases were analyzed for the control cells and transformed REFs.

The control primary RSFs showed a normal karyotype; i.e., 20 chromosomes, and X and Y. REFs that had been transformed by overexpression of C-MYC and Ha-RAS proteins showed a certain degree of chromosomal aberration (see Figure 5b).

Most unexpectedly, RSFs that overexpressed S18-2 showed an extremely high degree of chromosomal instability (Figure 5a and 5b). The diploid number of chromosomes varied between metaphases - from 70 to 79 in the analyzed metaphases (Figure 5a). The observed chromosomal instability might explain the development of tumors and aneuploidy exhibited by these cells, as noted above. The mechanisms by which overexpression of S18-2 protein affects chromosome duplication and segregation should be elucidated.

### Increased lipid droplet formation in cells that could give rise to tumors

**Figure 6:**
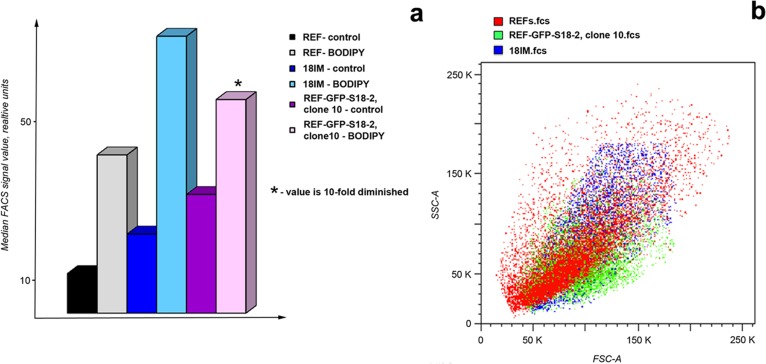
Lipid droplet formation in rat cells Cells were collected by trypsinization, washed once with PBS, and incubated with BODIPY 493/503. Samples were analyzed on a FACSAria II flow cytometer. The highest signal for lipid droplet staining was detected in the transformed cells of the REF–S18-2 clone 10 that produced tumors in SCID mice **a.** An overlay of side-scatter histograms for the studied cells is presented in panel **b.**

Increased lipid droplet formation has been observed both *in vivo* and *in vitro* in cancer cells (for review see [11]). To compare lipid droplet formation in the different cells, primary REFs and 18IM and REF–GFP–S18-2 (clone 10) cells were treated with BODIPY 493/503, which stains cellular lipid droplets. Flow cytometry showed that the strongest signal for lipid droplet staining was detected from transformed cells (REF–GFP–S18-2 clone 10), compared with 18IM cells and REFs (Figure 6a). Of note, the median fluorescence value was about 20-fold higher in transformed cells than in REFs. The side-scatter data led us to the same conclusion (see the overlay of side-scatter histograms for the studied cells in Figure 6b). REF–GFP–S18-2 clone 10, which had more lipid droplets than REFs and IM18 cells, had a smaller dimension compared with the other cells. This means that the number of lipid droplets was not influenced by cell size.

Here we have to mention, that primary REFs were unable to grow in soft agar, in contrast to 18IM and REF–GFP–S18-2 clone 10 cells, which showed anchorage-independent growth in soft agar. REF–GFP–S18-2 clone 10 cells gave rise to tumors in SCID mice.

These experiments are consistent with our other observations suggesting that overexpression of S18-2 cells causes cells to dedifferentiate into stem cells or to transform into malignant cells that are capable of forming tumors in SCID mice.

## DISCUSSION

To acquire the ability to divide indefinitely, cells must overcome so-called replicative senescence, a state of permanent growth arrest [12]. Primary rat fibroblasts are transformed after infection with retroviruses (e.g., HaMSV) when the Ha-RAS (viral RAS, v-RAS, P21^ras^) protein is markedly overexpressed [13]. *Ha-RAS* is a mutated cellular gene and its protein is a member of the small GTPase protein family. It is involved in growth receptor signaling through the ERK–MAPK pathway (reviewed in [14]). Introduction of *Ha-RAS* into REFs caused formation of foci in culture, but only a few colonies continued to proliferate. Because of chromosomal translocations, c-MYC protein is overexpressed in various human tumors, such as promyelocytic leukemia and Burkitt's lymphoma. However, only a small number of the growing transformed clones were obtained after introduction of *c-MYC* gene into primary REFs. By contrast, when both *c-MYC* and mutated *RAS* are introduced, REFs are easily transformed [15]. Thus, this is different from mouse cells, which can be easily immortalized spontaneously, and from human cells, which also require the inhibition of telomerase shortening upon cell division.

The next exciting chapter in the understanding of cell transformation started a few years ago when it was shown that mouse embryonic fibroblasts could be converted into induced pluripotent stem cells (iPSCs) *in vitro* by expression of the four genes *SOX2*, *OCT4*, *KLF4*, and *c-MYC* [16–18] or *MYCN* [19]. Rat fibroblasts could also be turned into iPSCs by the same four genes [20]. Similarly, human primary fibroblasts can be induced into stem cells by the same, or a slightly different, set of genes, such as *OCT4*, *SOX2*, *NANOG*, and *LIN28* [21]. In addition, expression of *OCT4* and *SOX2* in conjunction with valproic acid, a histone deacetylase inhibitor [22], can induce pluripotency in human fibroblasts. *SOX2* and *OCT4* can generate iPSCs from cord blood cells without valproic acid [23, 24].

It should be mentioned that generated iPSCs can develop into teratomas and other types of tumors in SCID and nude mice (reviewed in [25]). Many genes that induce pluripotency play an important role in cancer genesis and/or transformation. For example, a fusion gene comprising the N-terminus of Ewing sarcoma (*EWS*) gene and a portion of *OCT4*, a product of the chromosomal translocation t(6;22)(p21;q12), is considered to be a putative oncogene in bone tumors [26]. *SOX2* is localized on the long arm of chromosome 3 in the 3q26.33 region. The 3q26–27 locus is often amplified in solid tumors. *SOX2* is overexpressed in renal [27], prostate [28], colorectal [29], esophageal [30], and lung [31, 32] cancers. A high expression level of *LIN28* was found in embryonic carcinomas [33] and in epithelial ovarian cancers with poor prognosis [34]. Moreover, cells that express *LIN28* at a high level can produce tumors in nude mice. In such tumors, *c-MYC* is also overexpressed [35]. *LIN28* and *KLF4* expression is also elevated by MYCN in neuroblastomas [36].

We reported previously that overexpression of the mitochondrial ribosomal protein S18-2 alone leads to immortalization of primary REFs [8]. Here, we report that overexpression of S18-2 caused transformation of terminally differentiated RSFs. The transformed cells lost contact inhibition and became tumorigenic in SCID mice (Figure 1). These cells showed induction of stem cell maintenance markers, such as Sox2 and Oct4 (Figure 2). However, in contrast to 18IM cells, they did not lose mesodermal markers such as SMA and vimentin, although the vimentin signal was depolarized in transformed cells (Figure 3). E-cadherin and β-catenin, epithelial adhesion molecules, were detected in transformed cells (Figures 2 and 3). The expression of epithelial markers suggests that transformed rat fibroblasts are in the EMT. A recent study reported that hepatic stem cells coexpress mesenchymal and epithelial markers [37]. It is noteworthy that, during the course of mouse cell reprogramming by defined factors such as OCT4, SOX2, KLF4, and C-MYC, reprogrammed cells were found only in the E-cadherin-positive cell population [38]. Transformed RSFs surpassed the replicative senescence; although they were not induced to express stem cell properties as did 18IM cells.

Importantly, we found high levels of c-Myc and Klf4 expression at the RNA and protein levels in the clones that gave rise to tumors in experimental animals (Figure 2). High c-MYC expression is required for normal G_0_–G_1_ cell cycle progression [39–42]. Immortalized rat fibroblasts exhibit decreased proliferation when both alleles of the *C-MYC* gene are inactivated [40]. We speculate that, in transformed RSFs, c-MYC induction drives cells through the G_0_–G_1_ checkpoint. An additional argument is the induced chromosomal instability that was found in cells that gave rise to tumors (Figure 5).

As mentioned earlier, S18-2 protein binds RB and thus can inhibit the RB control over G_1_–S cell cycle progression [6, 7]. In immortalized 18IM cells, the nuclear S18-2 signal can be detected [8]. In the transformed RSFs (e.g., clone 6), the S18-2 signal was detected in both the cytoplasm and the nucleus. Upon overexpression of S18-2, REFs and RSFs showed increased telomerase activity (Figure 3), disturbance of the cell cycle (Figure 4), chromosomal instability (Figure 5), and increased lipid droplet content (Figure 6). Notably, increased lipid droplet biogenesis was detected in intestinal rat epithelial IEC-6 cells transformed by Ha-Ras [43]. It has been proposed that lipid droplets may be sites of *de novo* protein synthesis because mRNA, a proportion of ribosomal subunits, and translation-initiation factors have been detected in lipid droplets [44–46]. Increased translation and ribosomal synthesis suggest an increased proliferation rate of the transformed rat cells.

Taken together, our data lead us to speculate that S18-2 is a newly identified oncoprotein. The molecular mechanisms responsible for S18-2-induced transformation and the role of S18-2 protein in cancerogenesis require further elucidation.

## MATERIALS AND METHODS

### Cell transfections

Primary RSFs (2–4·10^5^ cells) were transfected with a plasmid, encoding GFP-fused S18-2 (GFP-S18-2) protein (described in [7]) or with vector, using Lipofectamine 2000 (Life Technologies, Carlsbad, CA, USA). Transfected cells were selected in Iscove's medium that contained 0.5 mg/ml G418 for 3 weeks. From a 7.5-cm-diameter Petri dish, 18 clones were selected and cultured in six-well plates. The fastest growing clones (numbers 3, 6, 13, and 17) were selected for further experiments. Clones were cultured for an entire observation period of >20 months.

No clones were observed upon vector transfection. 18IM cells [8] and REF-S18-2 clone 10 [9] derived by transfection of primary REFs by GFP-S18-2 were used as control cell lines.

### Cell culture and immunostaining

All cells were cultured in Iscove's medium that contained 10% FBS and appropriate antibiotics at 37°C. Before the experiment, cells were grown on cover slips in six-well plates. Cells were fixed in a mixture of cold methanol and acetone (1:1 at −20°C). Cells were rehydrated in phosphate-buffered saline (PBS) and stained.

An ethical permission to perform experiments with animals was obtained from Solna Court (290/11, 291/11 from 2011-08-31).

Tissue samples were paraffin embedded, and 5 μm sections were cut. In order to study protein expression by immunohistochemistry, the paraffin was dissolved in xylol and rehydrated with stepwise washing with EtOH in PBS (99%, 90%, 70% and 30% EtOH). Epitopes were exposed with hot citrate buffer (heating in the microwave oven for 5 min). EnVision™ Detection Systems Peroxidase/DAB (Dako, Glostrup, Denmark) was used for tissue section staining. The following primary antibodies were used: anti-smooth muscle actin (Dako); anti-vimentin (Dako); E-cadherin (Cell Signaling Technology, Danvers, MA, USA); and anti-S18-2 (clone 73-1 described in [7]). Rabbit anti-mouse and swine anti-rabbit FITC-conjugated (Dako) sera were used as secondary antibodies for immunostaining. Hoechst 33258 (Sigma-Aldrich, St. Louis, MO, USA) was added at a concentration of 0.4 μg/mL to the secondary antibody for DNA staining. The images were captured using a DAS microscope Leitz DM RB with a dual-mode cooled charge-coupled device (CCD) camera (C4880; Hamamatsu, Japan).

### Quantitative PCR (Q-PCR)

Total RNA was purified from all immortalized clones and control primary RSFs using a GeneJET™ RNA Purification kit (Fermentas GmbH, St. Leon-Rot, Germany). One microgram of total RNA was treated with DNase I (RNase-free) (Fermentas) to remove the genomic DNA in the RNA sample. One microgram of DNase-treated RNA was reverse transcribed to cDNA using a RevertAidTM Premium First-strand cDNA Synthesis kit (Fermentas). For Q-PCR, 20 μl of total reaction volume was prepared. MaximaTM SYBR Green/Fluorescein Q-PCR Master Mix (2μ) from Fermentas and 1 mM of each primer were used. The primer sequences for rat genes were: Klf4 (NM_053713) forward 5′-TTCTCCACGTTCGCGTCCGG-3′, reverse 5′-TCTCGCCAACGGTTAGTCGGGG-3′; C-Myc (NM_012603) forward 5′-TTGTTTTTTCGA TTTTAGAGAG-3′, reverse 5′-ATCCTTTCCCTTTCTATACAAT-3′; Nanog (NM_001100781) forward 5′-TTGGAACGCTGCTCCGCTCC-3′, reverse 5′-CGCCTGGCTTTCCCTAGTGGC-3′; Oct4 (NM_001009178) forward 5′-GGAGGGAT GGCATACTGTGGACCT-3′, reverse 5′-TCCTGGGACTCCTCGGGACTAGG-3′; Sox2 (NM_001109181) forward 5′-ACTAATCACAACAATCGCGGCGGC-3′, reverse 5′-GACGGGCGAAGTGCAATTGGGA-3′.

As an internal control for standardization, the expression of a gene encoding TATA-binding protein (Tbp, CH474033) was measured. The following primers were used: forward 5′-TTTCTTGCCAGTCTGGAC-3′, reverse 5′-CACGAACCACGGCACT GATT-3′.

The PCR cycling conditions were as follows: 10 min at 95°C, 40 cycles of 10 s at 95°C, and 1 min at 60°C. Applied Biosystems 7900 system software was used for the analysis. Ct values were determined for the internal control (Tbp) and the test genes at the same threshold level in the exponential phase of the PCR curves. The relative quantification (comparative Ct (ΔΔCt) method) was used to compare the expression levels of the test genes with the internal control. Dissociation curve analysis was performed after every run to check the specificity of the reaction. Three reactions (each in triplicate) were run for each gene, and the standard deviation was calculated.

### Western blotting

Cell lysates were prepared using NP40 lysis buffer (1% NP40, 150 mM NaCl, 50 mM Tris, pH 8) with protease inhibitor cocktail (Hoffmann La Roche Ltd, Basel, Switzerland). Protein concentration was measured using the Bradford protein assay and 20 μg of total protein was loaded in each well. SDS-PAGE gel electrophoresis was used to separate the proteins. Using wet transfer, proteins were transferred onto a nitrocellulose membrane. After transfer, the membranes were probed with mouse antibodies against smooth muscle actin, vimentin (Dako), c-MYC (Abcam Ltd, Cambridge, UK), and actin (Sigma-Aldrich), and rabbit antibodies against OCT4 (Novus Biologicals, Littleton, CO, USA), SOX2 (Abcam Ltd), and E-cadherin (Cell Signaling Technology). Secondary antibodies (horseradish peroxidase-conjugated anti-rabbit and anti-mouse IgG) were purchased from GE Healthcare Bio-Sciences AB (Uppsala, Sweden). Immunocomplexes were visualized using enhanced chemiluminescent reagents (GE Healthcare Bio-Sciences AB).

### Telomerase activity

A Quantitative Telomerase Detection (QTD) Kit from Biomax (Rockville, MD, USA) was used according to the manufacturer's protocol. The cell lysate containing 1 μg of the protein was added in each Q-PCR reaction. For the negative control, cell lysates were heated at 85°C for 10 min to inactivate telomerase activity, and an amount of the cell lysate equivalent to 1 μg of protein was added to the Q-PCR reaction. The control template (telomerase substrate oligonucleotide (TSR), 0.5 amole/μl) was added in serial dilutions to produce a standard curve. One-to-five dilutions were prepared starting from 0.5 amole/μl to 0.00016 amole/μl. 2X QTD Premix was used for Q-PCR, and the total volume of the reaction was 25 μl. The Q-PCR cycling conditions were as follows: 20°C for 25 min, 95°C for 10 min, and 40 cycles at 95°C for 30 s, 60°C for 30 s, and 72°C for 30 s. The threshold cycle was determined in the exponential phase of the Q-PCR curves, and dissociation curve analysis was performed after every run. A standard curve was obtained for the TSR molecule numbers, which were determined for each cell line from the standard curve.

### Cell cycle analysis by flow cytometry

One million cells were labeled with BrdU (bromodeoxyuridine, 30 μM) for 30 min at 37°C, harvested by trypsinization, and fixed in 75% ethanol in PBS overnight at 4°C. Cells were then treated with pepsin (1 mg/ml in 30 mM HCl) for 30 min at 37°C followed by 2 M HCl for 15 min. The cells were labeled with mouse anti-BrdU primary antibody (Becton Dickinson (BD), San Jose, CA, USA) and FITC-conjugated rabbit anti-mouse secondary antibody (Dako), and counterstained with propidium iodide (25 μg/ml in PBS). 10 000 cells were analyzed, using a FACScan flow cytometer (BD), and the percentage of cells in each phase of the cell cycle was determined, using CellQuest software (BD).

### Karyotyping

20 hours after fresh medium was added, it was replaced with IMDM that contained 0.5 ml of Colcemid (concentration of stock solution was 10 μl/ml, GIBCO, NY, USA). Cells were incubated in this medium for 4 hours. Cells, collected by centrifugation, were re-suspended in 0.075 M KCl and incubated for 1 hour, as described in [47]. A freshly prepared mixture of methanol and acetone (3:1) was used to fix cells and after the repeated washing and centrifugation the metaphases were prepared on glass slides. They were dried at the room temperature for 2 weeks. Slides were incubated in SSC at 62°C for 2 hours with the following trypsinization (0.025% trypsin for 15-30 sec) and stained with Giemsa solution.

### Quantification of cellular lipid droplets

Cells were collected by trypsinization, washed once with PBS and incubated with BODIPY 493/503 (Molecular Probes, Invitrogen) for 15 min at the room temperature in the dark. Stained cells were washed twice with PBS and re-suspended in PBS then. Samples were analyzed by FACSAria II flow cytometer (BD Biosciences). To allow comparison of the different cell lines, gain for fluorescence PMTs was kept the same for all the measurements.
